# Editorial: Advancements in solid tumor immunotherapy: enhancing efficacy and overcoming resistance

**DOI:** 10.3389/fcell.2026.1814863

**Published:** 2026-03-31

**Authors:** Yi Jiang, ShaoDong Wang, Leqi Zhou, Tao Zhang, Haoran Feng, Tao Zhang

**Affiliations:** 1 Department of General Surgery, Ruijin Hospital, Shanghai Jiao Tong University School of Medicine, Shanghai, China; 2 Department of General Surgery, Changhai Hospital, Second Military Medical University, Shanghai, China; 3 Human Oncology and Pathogenesis Program (HOPP), Memorial Sloan Kettering Cancer Center (MSK), New York, NY, United States; 4 Shanghai Institute of Digestive Surgery, Ruijin Hospital, Shanghai Jiao Tong University School of Medicine, Shanghai, China; 5 Institute of Medical Robotics, Ruijin Hospital, Shanghai Jiao Tong University School of Medicine, Shanghai, China

**Keywords:** immunotherapy, neoadjuvant chemoimmunotherapy, NK cells, patient-derived organoids, resistance mechanisms, tumor microenvironment

## Introduction

Immunotherapy has reshaped the treatment landscape of many cancers, yet durable benefit in solid tumors remains limited to a subset of patients. Primary resistance, adaptive immune escape, and toxicity constraints continue to restrict broader impact. Increasingly, it is clear that response is not determined solely by tumor-intrinsic alterations, but by a dynamic tumor ecosystem—immune, stromal, metabolic, and treatment-induced states—evolving over time. This Research Topic, Advancements in Solid Tumor Immunotherapy: Enhancing Efficacy and Overcoming Resistance, assembles 14 contributions spanning enabling platforms, mechanistic insights, biomarkers and prediction tools, and clinical/translational evidence across multiple tumor types. Collectively, these articles converge on a practical roadmap: (i) build more faithful human-centered models, (ii) decode ecosystem-level drivers of immune dysfunction and stromal immunosuppression, and (iii) deploy mechanism-guided combinations supported by accessible, clinically actionable stratification.

## Bridging the translational gap: from models to “living biomarkers”

A persistent bottleneck in solid tumor immunotherapy is the mismatch between preclinical systems and patient reality. The review on patient-derived organoids (PDOs) positions PDO-based platforms as a transformative bridge to precision immuno-oncology, emphasizing immuno-PDO (iPDO) strategies that either reconstitute immune components (e.g., co-culture with exogenous immune cells) or preserve native tumor immune context (e.g., air–liquid interface or patient-derived organotypic tumor spheroids) (Ni et al.). Framed as a “problem–solution” approach, the article highlights how iPDOs can (1) deconvolute immunosuppressive tumor microenvironments, (2) function as “living biomarkers” for response prediction, (3) help unravel resistance via multi-omics, and (4) enable high-throughput screening for personalized combinations—especially when integrated with bioengineering and AI.

Complementing this platform theme, the review on pancreatic exocrine organoids and spheroids underscores the value of 3D systems for drug screening and precision medicine in a disease context where heterogeneity and therapeutic resistance are especially prominent (Tan et al.). Together, these contributions reinforce that improving immunotherapy outcomes depends not only on new drugs, but on predictive, scalable human models that can guide rational combination design and patient selection.

## Ecosystem-level resistance: stromal control nodes and immune dysfunction

Resistance is often a systems property emerging from tumor–stroma–immune interactions. An original cross-cancer single-cell analysis in this Research Topic charts ecosystem dynamics under immunochemotherapy and identifies a stromal axis in which S100A4 in fibroblasts associates with enhanced PD-L1 expression in tumor cells, implicating fibroblast programs and microenvironmental remodeling as actionable levers for overcoming immunosuppression (Yang et al.). This work aligns with the broader view that effective combination immunotherapy should target not only malignant cells and checkpoints, but also the non-malignant compartments that shape immune exclusion and exhaustion.

A complementary perspective is provided by the review on Kuznetsova et al., which emphasizes that TME properties—particularly extracellular matrix (ECM) composition and biophysical constraints (e.g., stiffness, porosity), alongside metabolic features such as hypoxia and acidity—can limit NK cell infiltration, persistence, and cytotoxic function in solid tumors. By focusing on ECM and mechanotransduction as therapeutic targets, the review highlights interdisciplinary opportunities (engineering, materials science, nanotechnology) to remodel the TME into a more permissive niche for effective innate and adaptive immune function.

Disease-specific resistance is further illustrated by the review on uveal melanoma, which synthesizes barriers to checkpoint blockade and highlights emerging strategies to enhance efficacy and overcome resistance (Song et al.).

## Tumor-intrinsic vulnerabilities and treatment intersections

Several articles underscore how tumor-intrinsic programs intersect with response and can be exploited for sensitization strategies. The original research on PTEN and melanoma radiosensitivity reports that PTEN overexpression inhibits the DNA-PKcs axis, reduces NHEJ-mediated rapid repair, and increases persistent DNA damage signaling (γ-H2AX), translating into enhanced radiosensitivity *in vitro* and in xenograft settings (Zhou et al.). Importantly, the study frames PTEN as both a potential biomarker for radiotherapy response prediction and a target for sensitization interventions—reinforcing the growing interest in rational radio–immunotherapy designs, where DNA damage pathways may influence immunogenicity and immune-mediated clearance.

In addition, the contribution on microtubule regulation and dynamic instability points toward quantitative characterization of cell-state vulnerabilities linked to drug resistance, with potential relevance to immune cell function and cellular therapies (Matov). Together, these works reflect a unifying theme: resistance can be rooted in cell-state plasticity and stress-response circuitry, suggesting multi-node interventions may outperform single-target strategies.

## Prediction and stratification: making treatment selection actionable

As immunotherapy expands into neoadjuvant and perioperative settings, the need for practical, accessible stratification tools becomes urgent. The original research developing a nomogram to predict pathological complete response (pCR) after neoadjuvant chemoimmunotherapy in resectable NSCLC addresses this gap using routine clinical variables (Liu et al.). In a retrospective cohort of 179 stage IIB–IIIB patients, the model identifies independent predictors including histology, family history, smoking cessation duration, age, and number of treatment cycles, achieving modest discrimination (reported AUC ∼0.71) with internal validation and decision curve analysis, while noting the need for external validation and improved generalizability. This work exemplifies an important translational direction: complementing biomarker-based approaches with resource-feasible tools that can inform surgical planning and individualized decisions.

## Pragmatic evidence and rare-tumor insights

This Research Topic also includes evidence closer to real-world implementation. The retrospective comparison of domestic versus imported immune checkpoint inhibitors in neoadjuvant chemoimmunotherapy for esophageal squamous cell carcinoma provides pragmatic information relevant to settings where multiple PD-1 options are accessible and where cost, availability, and local practice patterns matter (Chen et al.).

Two case reports further illustrate how molecular and pathological features can extend immunotherapy benefit into rare contexts. A report of MS-stable/TMB-high pleomorphic liposarcoma describes a meaningful response to pembrolizumab in the setting of postoperative liver metastases, supporting the hypothesis that TMB-high may retain predictive value even in sarcoma subsets where ICI efficacy is variable (Miwa et al.).

Another case report of advanced pulmonary epithelioid hemangioendothelioma describes radiologic improvement and disease stabilization with sintilimab combined with chemotherapy, in a tumor characterized by limited standard options, highlighting how PD-L1 status and careful translational characterization can motivate future systematic evaluation in rare diseases (Jing et al.).

## Broadening the therapeutic toolbox: targets and combination backbones

Beyond PD-1/PD-L1, the Research Topic includes reviews that expand the target landscape and combination logic. The CD70 review summarizes advances and challenges in targeting the CD70/CD27 axis across modalities (Wu et al.), while the mini review on bladder cancer immunotherapy integrates BCG, checkpoint blockade, viral vectors, nanotechnology, and CAR-based strategies—reflecting a broader trajectory toward multi-modal regimens tailored to disease context and patient biology (Spirito et al.). In parallel, the gemcitabine resistance review emphasizes that overcoming treatment failure often requires integrating immune strategies with optimized conventional backbones and delivery innovations (Zhang et al.).

## Outlook

Across these 14 contributions, three consistent directions emerge. First, the field must continue investing in human-centered, ecosystem-aware platforms—especially iPDO systems—to narrow the translational gap and accelerate functional testing of combinations and sequencing. Second, overcoming resistance will require targeting microenvironmental control nodes, including stromal programs and ECM/biophysical barriers that constrain immune infiltration and function, alongside tumor-intrinsic vulnerabilities such as DNA repair circuitry and cell-state plasticity. Third, successful clinical implementation will increasingly depend on biomarker- and prediction-guided selection, combining pragmatic decision tools (e.g., nomograms) with molecular and ecological markers to individualize therapy and minimize overtreatment.

We hope this Research Topic provides a coherent reference for researchers and clinicians seeking to enhance immunotherapy efficacy in solid tumors while confronting—and ultimately overcoming—the diverse resistance mechanisms that limit durable benefit.

